# Supplementary material for: Visualization of the Dynamics of Photoinduced Crawling Motion of 4-(Methylamino)Azobenzene Crystals via Diffracted X-ray Tracking
Source: Int J Mol Sci. 2023 Dec 14;24(24):17462. doi: 10.3390/ijms242417462 (PMC10744157; doi:10.3390/ijms242417462)
Supplement: Supplementary file 1 [file ijms-24-17462-s001.zip › ijms-2719758-supplementary.pdf]

Supporting Information

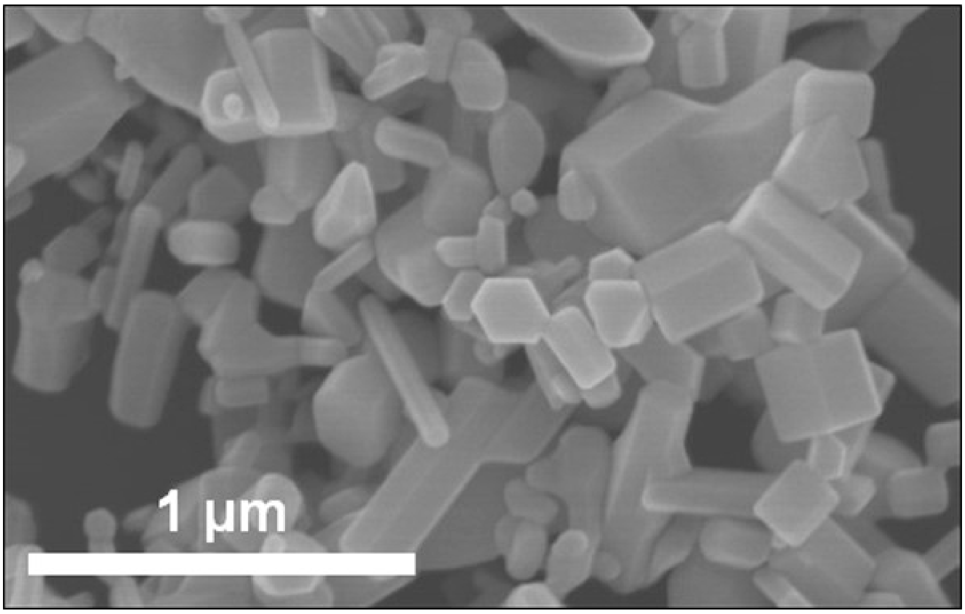

Figure S1. A scanning electron microscopy image of the zinc oxide nanoparticles used in the experiments.

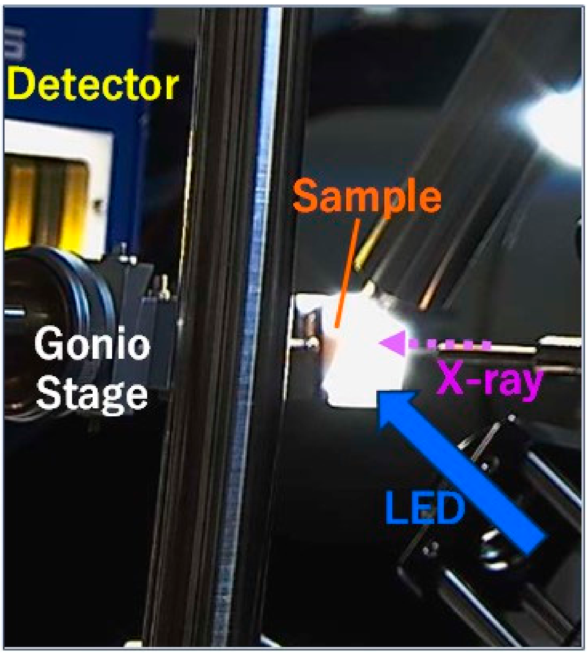

Figure S2. A photographic image of the XRD measurement setup. Diffraction X-rays transmitted through the sample were detected.

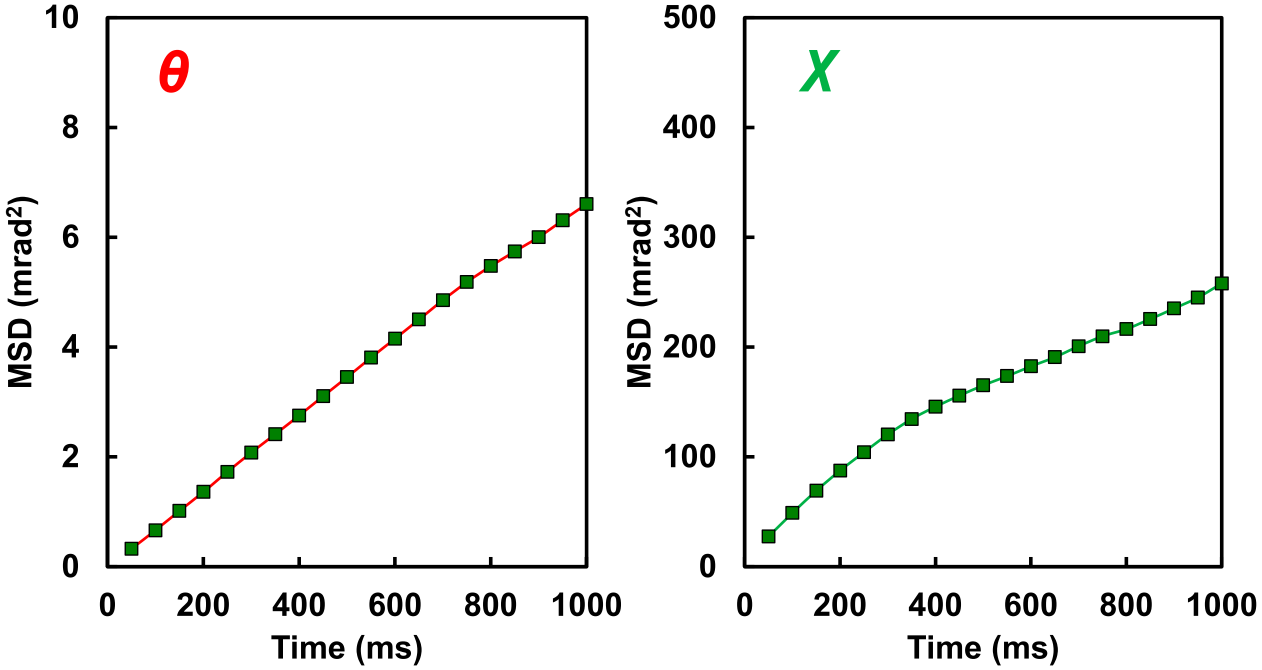

Figure S3. MSD curves excluding the rotational displacement at the moment when the 4-MAAB crystalline film completely melted at the light intensity 200 mW cm^{-2}.

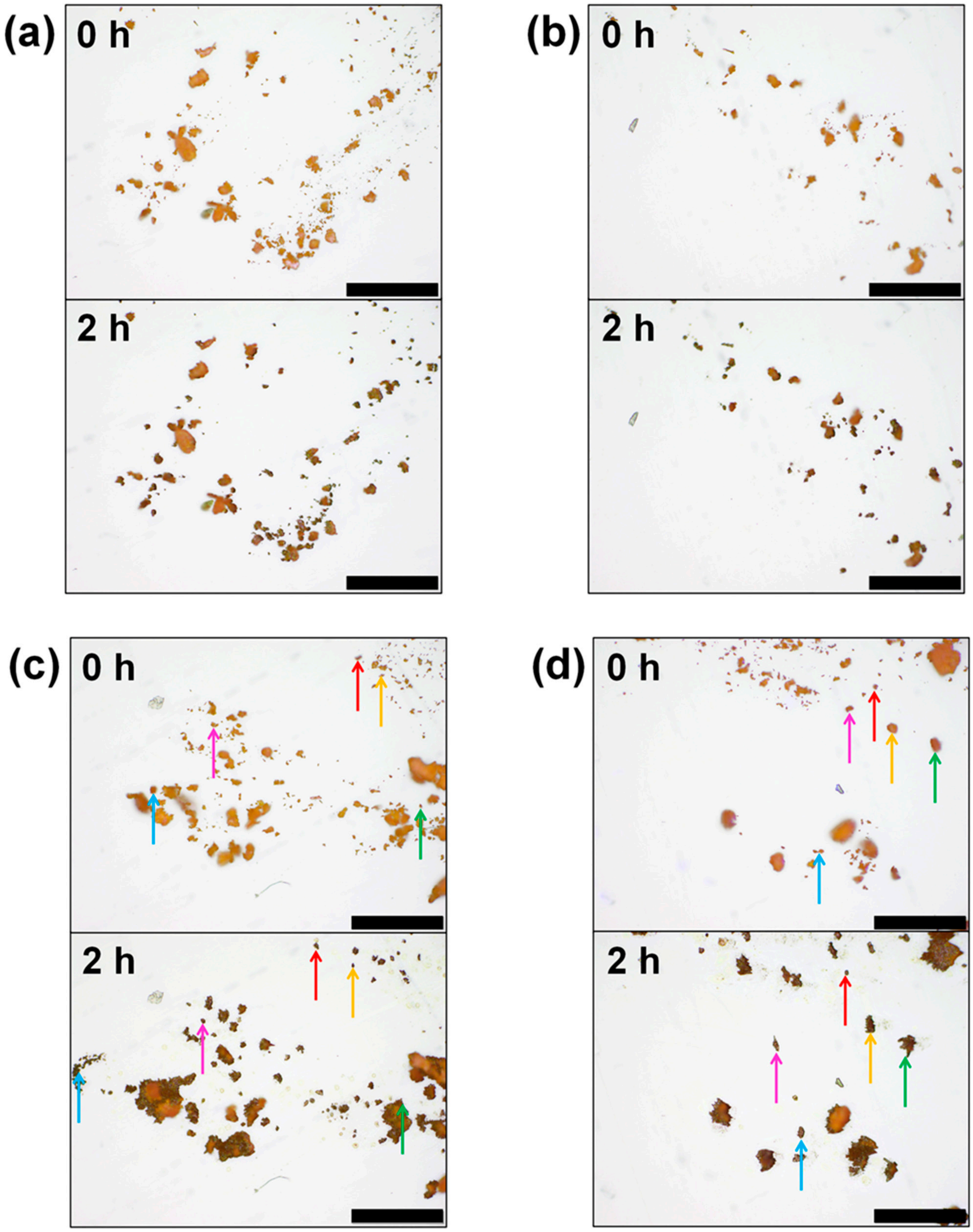

Figure S4. (a-d) Optical microscope images of 4-MAAB crystals on polyimide films before and after light irradiation at the light intensity (a) 50, (b) 100, (c) 200, and (d) 300 mW cm^{-2} (scale bar: 200 μm). Blue LED light was irradiated for 2 h. The arrows shown in Figure 1c, d point to 4-MAAB crystals that exhibited photo-induced crawling motion.

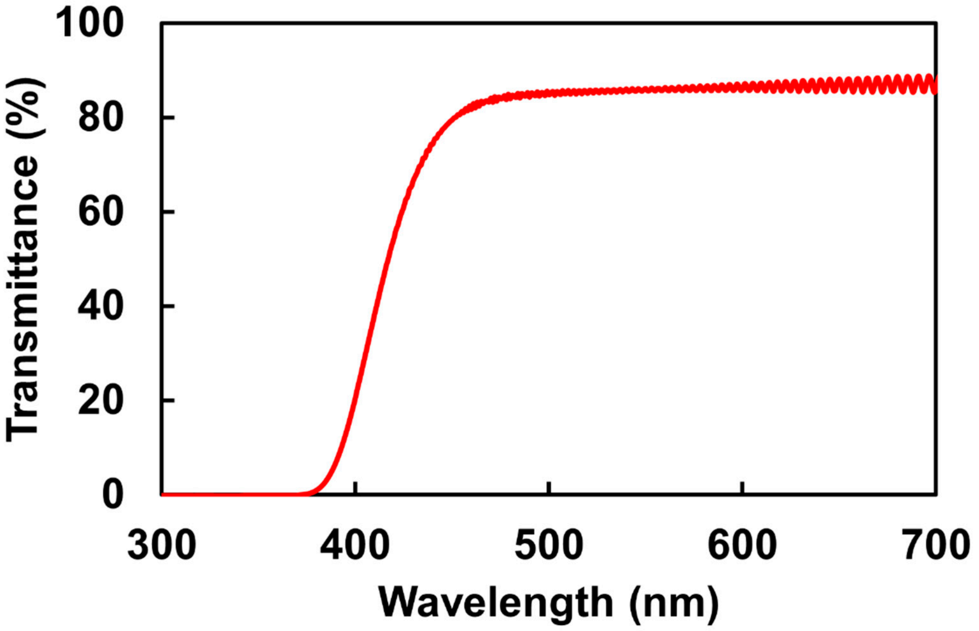

Figure S5. Transmittance spectrum of the polyimide film used in the experiment.

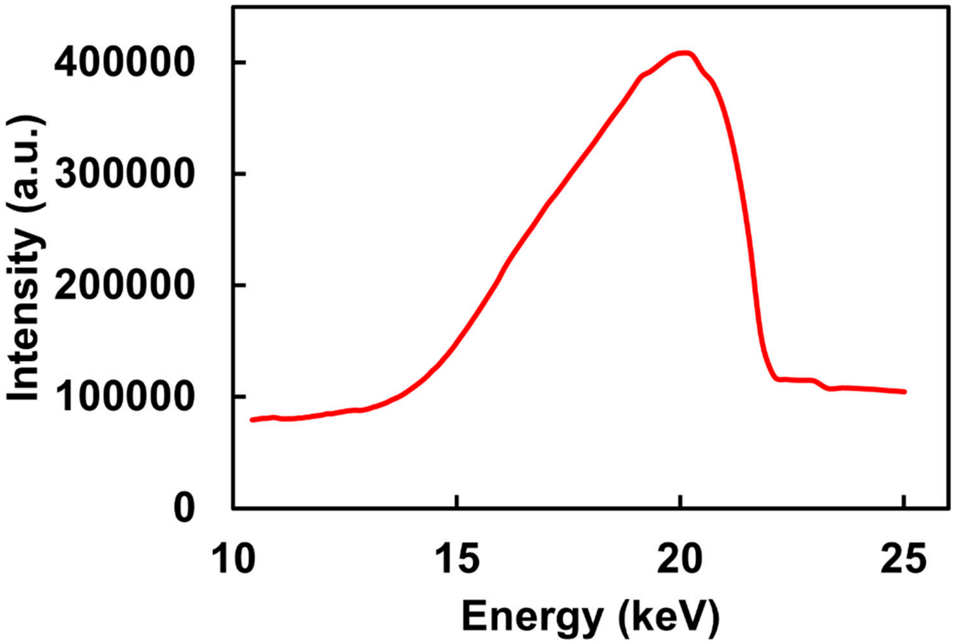

Figure S6. An intensity spectrum of the white X-ray.

Movie_S1.mp4

Movie S1. Time-resolved XRD pattern of 4-MAAB powder crystals containing ZnO nanoparticles under 200 mW cm^{-2} blue light irradiation. (Video fast-forwarded 5 × compared to real time; MP4)

Movie_S2.mp4

Movie S2. Melting behavior of a 4-MAAB crystalline thin film containing ZnO nanoparticles under under 200 mW cm^{-2} blue light irradiation. (Video fast-forwarded 5 × compared to real time; MP4)

Movie_S3.mp4

Movie S3. Time-resolved XRD pattern of a 4-MAAB crystalline thin film containing ZnO nanoparticles under 200 mW cm^{-2} blue light irradiation. (Video fast-forwarded 5 × compared to real time; MP4)
